# The concept of negative pressure wound therapy (NPWT) after poststernotomy mediastinitis – a single center experience with 54 patients

**DOI:** 10.1186/1749-8090-4-5

**Published:** 2009-01-12

**Authors:** Ina C Ennker, Anita Malkoc, Detlef Pietrowski, Peter M Vogt, Juergen Ennker, Alexander Albert

**Affiliations:** 1 Mediclin Herzzentrum Lahr/Baden, Abteilung Herzchirurgie, Hohbergweg 2, 77933 Lahr, Germany; 2 Hannover Medical School, Klinik für Plastische, Hand- und Wiederherstellungschirurgie, Carl-Neuberg-Str. 1, 30625 Hannover, Germany; 3 Medical University Vienna, Department for Womens Health, Austria

## Abstract

Deep sternal infections, also known as poststernotomy mediastinitis, are a rare but often fatal complication in cardiac surgery. They are a cause of increased morbidity and mortality and have a significant socioeconomic aspect concerning the health system. Negative pressure wound therapy (NPWT) followed by muscular pectoralis plasty is a quite new technique for the treatment of mediastinitis after sternotomy. Although it could be demonstrated that this technique is at least as safe and reliable as other techniques for the therapy of deep sternal infections, complications are not absent. We report about our experiences and complications using this therapy in a set of 54 patients out of 3668 patients undergoing cardiac surgery in our institution between January 2005 and April 2007.

## Background

Poststernotomy mediastinitis or deep sternal infection (DSI), is a serious cause of postoperative morbidity and mortality in cardiac surgery patients. Its incidence is reported to be between 1% and 3% and complications are associated with a significant mortality between ranging 10% and 25% [1-3]. A wide range of wound-healing strategies have been established until now for treatment of this often devastating complication. Negative pressure wound therapy was first introduced by Argenta and Morykwas [4,5] and has become a widely used, efficacious and reliable method for managing different types of open wounds (e.g. abdomen, head, neck) after infection. In cardiac surgery it is frequently applied for treating patients suffering from deep sternal wound infections. Although greater randomized controlled studies are missing, it is generally suggested that it decreases morbidity and mortality associated with DSI after thoracotomy. Recent publications have shown promising results for this method [6,7]. However, the strategy for surgical wound debridement is not standardised and varies significantly from surgeon to surgeon.

We analyzed our experiences and complications with 54 patients in our institution suffering from DSI between January 2005 and April 2007 out of 3668 patients undergoing cardiac surgery procedures. The 54 patients were treated by NPWT and a subsequent pectoralis muscle plasty for secondary closure. The observed complications during their hospital-stay until discharge are reported.

## Methods

We present an analysis of all consecutive patients suffering from DSI after cardiac surgery between January 2005 and April 2007 in our centre. All of these patients received NPWT therapy for wound closure. They were suffering from mediastinitis, partly with the involvement of the sternum. Demographical data, general treatment and patient outcomes were collected. Information was retrieved according to hospital medical ethic committee. Diagnosis of DSI is conducted by at least one of the following criteria: Isolation of a germ with infectious potential, demonstration of wound infection during operational procedures or purulent discharge accompanied with temperature above 38°C. The management of cardiac patients referred with sternal wound complications were described previously [6]. The classification of DSI is based on the international classification provided by El Oakley and Wright [8]. The onsets of DSI as well as the existence of specific risk factors for wound infections are incorporated. The allocation system is presented in table 1.

**Table 1:** Classification of mediastinitis in patients undergoing cardiac surgery.

Class	Description
Type I	Mediastinitis presenting within 2 weeks after operation in the absence of risk factors

Type II	Mediastinitis presenting at 2 to 6 weeks after operation in the absence of risk factors

Type IIIA	Mediastinitis type I in the presence of one or more risk factors

Type IIIB	Mediastinitis type II in the presence of one or more risk factors

Type IVA	Mediastinitis type I, II, or III after one failed therapeutic trial

Type IVB	Mediastinitis type I, II, or III after more than one failed therapeutic trial

Type V	Mediastinitis presenting for the first time more than 6 weeks after operation

According to El Oakley and Wright [8] mediastinitis is defined as wound infection associated with sternal osteomyelitis with or without infected retrosternal space. Incremental risk factors for mediastinitis are diabetes, obesity, and the requirement of immunosuppressive agents. Failed therapeutic trial includes any surgical intervention with intent to treat mediastinitis.

## Results

**Figure 1:**
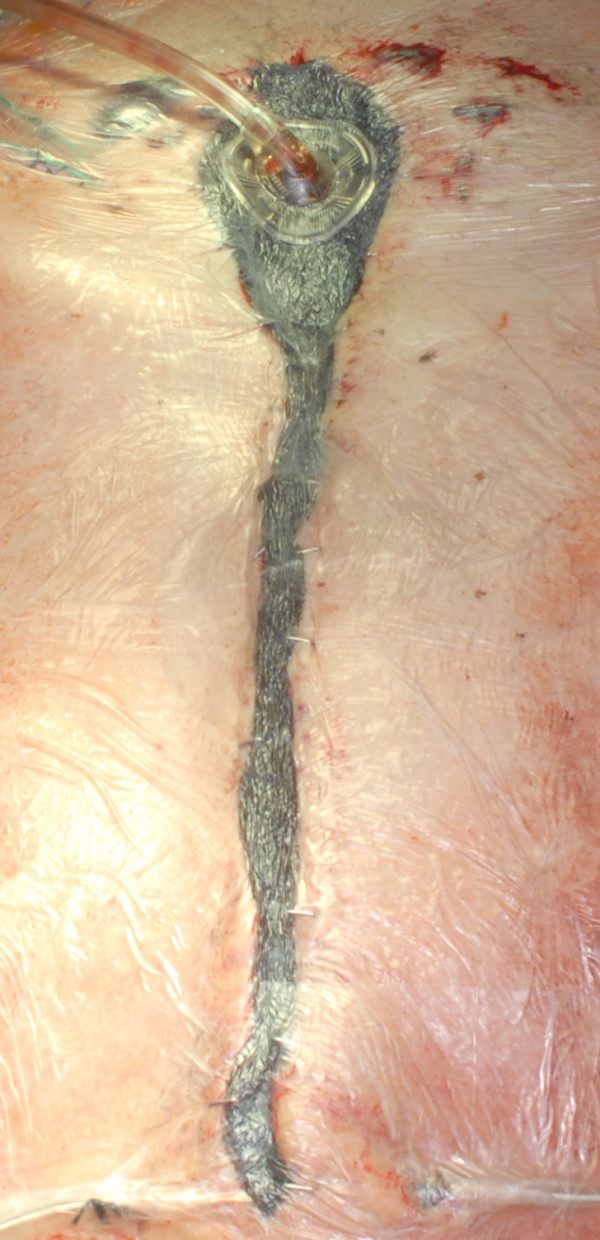
**Patient after bypass operation and deep sternal wound infection treated in our hospital**. The patient received NPWT before surgical approach for cleaning of the wound.

**Figure 2:**
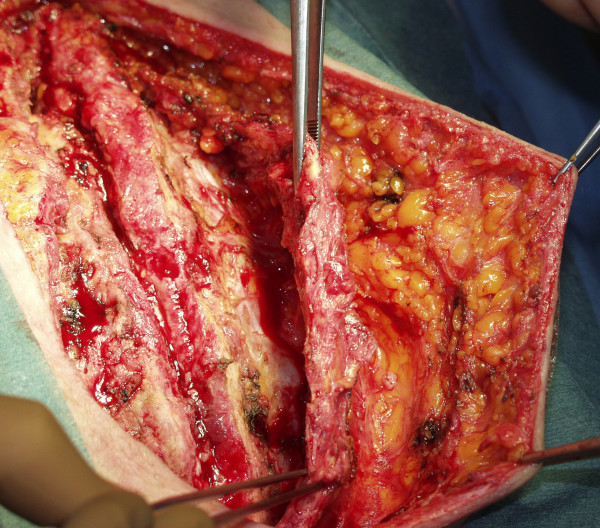
**The figure shows the wound prior to refixation with pectoralis muscle already prepared for coverage of the sternum (a) and with the pectoralis plasty in place**.

**Figure 3:**
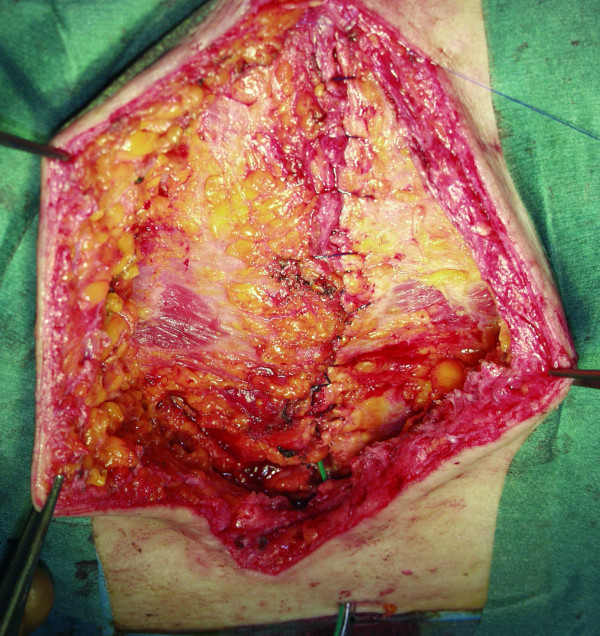
**The figure presents the complete closure of the wound after NPWT therapy and pectoralis plasty**.

There were 54 patients suffering from DSI in this period. All patients were treated with NPWT therapy (see figure 1 and 2) and subsequent pectoralis muscle plasty for secondary wound closure. The result of successful wound closure after pectoralis plasty is presented in figure 3. The most frequently detected germ was staphylococcus epidermis (37.8%) followed by enterococcus faecilis (18.9%), staphylococcus aureus (13.5%) and others (16.3). In 13.5% of all patients no bacteria could be detected.

According to the classification system by El Oakley et al. [8] we classified the patients into seven groups. Most of the patients encompassed a mediastinitis class IIIB (33.3%) followed by class IIIA (25.9%). Less frequently we observed mediastinitis class II (18.5%), class V (11.1%), class IVB (5.6%), class I (3.7%) and class 4A (1.9%).

Patient-related factors differ significantly between patients developing DSI and those not developing DSI (table 2): They were more often male, had higher body-weight, a higher prevalence of diabetes and chronic obstructive pulmonary disease (COPD). Notably, preoperative white blood cell count and CRP-levels were equal in both groups. Whereas intraoperative data did not differ significantly from other patients, those patients who developed DSI showed higher complication rates in the early postoperative course before detecting DSI (table 3): We noticed a higher rate of reintubation and rethoracotomy, a lower haemoglobin level and higher levels of white blood cell counts in these cases. All of these patients had a prolonged stay on ICU.

**Table 2:** Preoperative patient characteristics of patients developing DSI and control group

Patient characteristics, preoperative	Mediastinitis group	Control group	P-value of univariate comparisons
Male gender	43 (81%)	3189 (69%)	< 0,05

Age [years]	67,9 ± 1,4	67,8 ± 0,15	n.s.

Body height [cm]	168,7 ± 1,2	170 ± 0,12	n.s.

Body weight [kg]	88,9 ± 2,1	80,7 ± 0,2	< 0,001

Body-mass-index	31,3 ± 0,6	27,9 ± 0,07	< 0,001

Diabetes, oral	14 (26%)	851 (18%)	< 0,05

Diabetes, insulin-dependent	12 (22%)	549 (12%)	< 0,05

Ejection fraction [%]	54 ± 3,7	58 ± 0,4	n.s.

Hemoglobin, preoperative [g/dl]	13,5 ± 0,2	13,3 ± 0,02	n.s.

Hematocrit, preoperative [%]	40,3 ± 0,7	39,4 ± 0,1	n.s.

Leukocytes, preoperative [k/UL]	8,4 ± 0,7	7,8 ± 0,1	n.s.

Serum creatinine, preoperative [mg/dl]	1,2 ± 0,1	1,2 ± 0,01	n.s.

Creatinine kinase MB, preoperative [U/l]	21 ± 16,1	28,7 ± 2,0	n.s.

Chronic obstructive pulmonary disease	20 (38%)	918 (20%)	<0,01

Reduced ejection fraction	24 (44%)	1620 (34%)	P = 0,11

EuroSCORE	5,7 ± 0,4	5,9 ± 0,05	n.s.

CRP, preoperative [mg/l]	16,5 ± 3,9	11,4 ± 0,4	P = 0.19

According to El Oakley and Wright [8] mediastinitis is defined as wound infection associated with sternal osteomyelitis with or without infected retrosternal space. Incremental risk factors for mediastinitis are diabetes, obesity, and the requirement of immunosuppressive agents. Failed therapeutic trial includes any surgical intervention with intent to treat mediastinitis.

**Table 3:** Intra- and postoperative patient characteristics of patients developing DSI and control group

Indicators of course after major cardiac surgery	Mediastinitis group	Control group	P-value of univariate comparisons
Double internal mammary arteries	13 (24%)	1112 (23%)	n.s.

Pump time [min]	94,5 ± 7,3	81,2 ± 0,8	= 0,06

Aortic clamp time	68 ± 4,9	57 ± 0,51	<0,05

Operation time	207 ± 8,1	192,2 ± 0,9	= 0,06

Postoperative stay [days]	33 ± 1	7,0 ± 0,1	< 0,001

Atrial fibrillation, postoperative	30 (56%)	2042 (43%)	P = 0.06

Reintubation	19 (35%)	193 (4%)	< 0,001

Pleural effusions	24 (44%)	1998 (42%)	n.s.

Rethorakotomy	7 (13%)	113 (2,3%)	< 0,001

Hemoglobin, 6 h postoperative [g/dl]	10,5 ± 0,2	10,4 ± 0,16	n.s.

Hemoglobin, discharge [g/dl]	10,5 ± 0,2	11,2 ± 0,02	< 0,001

Erythrocytes, minimum during stay [g/dl]	2,8 ± 0,05	3,0 ± 0,01	< 0,001

Leukocytes, 6h postoperative [k/UL]	10,7 ± 0,8	10,0 ± 0,1	n.s.

Leukocytes, maximum postoperative [k/UL]	17,6 ± 1,1	14,3 ± 0,1	< 0,01

Serumcreatinine, postoperative [mg/dl]	2,4 ± 0,15	1,6 ± 0,02	< 0,001

Higher serum sodium postoperative [mmol/l]	145,9 ± 0,5	143,7 ± 0,05	< 0,001

The predictive models identified well-known pre- and postoperative risk factors for development of DSI (tables 2 and 4). In order to focus on intraoperative factors we compared the predictive and observed incidence of DSI for different types of operation (table 5) and also the surgeons (table 6), but were not able to find any intraoperative risk factors. Notably, the use of bilateral mammary arteries did not increase the risk for DSI.

**Table 4:** Multivariate risk factors for postoperative mediastinitis

Term	Estimate	Std Error	P-value	Odds Ratio
Intercept	6,01	1,48	< 0,001	

Chronic obstructive pulmonary disease	0,35	0,15	0,02	2,01

Rethoracotomy (other than wound infection)	0,30	0,25	0,23	1,82

Reintubation	1,14	0,17	< 0,001	9,79

Higher Body mass index	-0,14	0,02	< 0,001	0,00

More red cell package postop	-0,03	0,02	0,19	0,10

Lower haemoglobin at discharge	0,19	0,11	0,09	7,15

Male gender	-0,45	0,19	0,02	0,41

Higher creatinine postoperative	-0,16	0,09	0,08	0,14

**Table 5:** No significant differences between types of operation were observed (ANOVA)

Type of Surgery	N	Expected risk for mediastinits [%]	Observed incidence of mediasitnitis [%]	Observed/Expected
On-PCAB	1671	1,1	1,6	1,4

O(ff)-PCAB	917	1,0	0,4	0,4

Valve&CABG	710	1,3	1,7	1,3

Aortic surgery	229	1,2	0,4	0,1

Isolated Valves	2582	1,1	1,2	1,0

Double valves	76	1,1	1,3	4,2

Re-do valves	106	1,5	0,9	0,2

Miscellaneous	174	1,1	1,7	1,7

**Table 6:** No significant differences between the surgeons were observed (ANOVA)

Surgeons	N	Expected risk for mediastinits [%]	Observed incidence of mediasitnitis [%]	Observed/Expected
Surg. A	331	1,2	1,2	1,0

Surg. B	294	1,0	1,7	1,6

Surg. C	746	1,1	0,7	0,6

Surg. D	685	1,3	1,3	1,0

Surg. E	205	1,4	0,0	0,0

Surg. F	921	1,0	1,0	0,9

Surg. G	576	1,2	1,4	1,1

Trainees	1031	1,1	1,2	1,1

The detection of DSI and subsequent treatment with NPWT of the patients is clearly prolonged: the period of time that patients stayed in hospital ranged from 22 days up to 185 days (median 64.5 days). The time period from the date of operation until detection of mediastinitis ranged from 5 days to 155 days (19 days). Time period from the start of the NPWT therapy until date of discharge from hospital ranged from 7 days until 99 days with a median at 35 days.

In addition complications occurred during the period after detection of mediastinitis until discharge from hospital (table 7): The most common complication was atrial fibrillation followed by respiratory failure, other infections than sternal infections and rethoracotomy. In 74.1 percent of all patients no complication was reported (table 6).

**Table 7:** Complications occurred during NPWT

Complication	Number/	Percent
Instable sternum	1	1.9

Infection other than Mediastinitis	2	3.7

Respiratory failure	4	7.4

Atrial fibrillation	5	9.3

Re-Thoracotomy	1	1.9

Rupture of the right ventricle	1	1.9

No complication	40	74.1

Pitfalls based mainly on the technical and surgical handling during the procedure are the use of pressure lower than 120 mm Hg that causes dismantling of the foil and the need of subsequent debridement and revision of the wound. In three cases we had to redo soft tissue reconstruction unilateral, and patients necroses of the pectoralis muscle because of loosening of muscular sutures. In four cases the vacuum worked insufficiently because of a backflow of liquids into the tubes connecting the sealed wound with the vacuum pump. The most serious complication was in one patient, who died due to rupture of the right ventricle. After an uneventful course he abruptly developed circulatory collapse after coughing with massive blood loss from the wound at the seventh day after NPWT. Emergency thoracotomy revealed right ventricular wall rupture by sharp sternal edges due to sternal instability.

## Comments

Although much progress has been made in the treatment of postoperative infections, poststernotomy mediastinitis or DSI is still a devastating complication of cardiac surgery. Numerous studies have proven that an increase in morbidity and mortality and a decrease in long-term survival are associated with mediastinitis after cardiac surgery. Since it is also coupled with a longer hospital-stay and an increment of nursing care, it has a considerable economic impact to the health system [9,10].

In the classification system by El Oakley et al. only diabetes, obesity and immunosuppressive therapies are risk factors for this complication. Using this classification system one third of our patients had no risk factor and 70 percent had one or more risk factors. Other risk factors found in the literature for the development of a postoperative mediastinitis include higher age, malnutrition, smoking etc. [2]. Beside the management of metabolic factors (especially diabetes), other risk factors identified in our study e.g. COPD and obesity may also reflect the higher mechanical forces present in these patients leading to instability of the sternum. In these cases special techniques of sternum closures e.g. the use of double armed wires are an alternative. Although we could not prove an impact of the individual surgeon on the incidence of DSI, the fact that one surgeon had no incidence of DSI in more than 200 patients may also reflect an adequate technique of chest opening and closure. The careful use of electrocautery, the complete closure of the fascia, the use of single sutures of the fascia and the skin in exposed patients may help to prevent DSI. It was obvious in our study how the incidence of DSI depend on major postoperative complication like reintubation and rethoracotomy.

Treatment of DSI using NPWT is described since 10 years [11]. Since then more than 400 cases have been published describing NPWT as suitable therapy for deep sternal wounds after cardiac surgery. NPWT therapy is known to increase the capillary diameter, the blood flow velocity and to stimulate angiogenesis and endothelial cell proliferation and thereby promoting tissue proliferation and wound closure [12]. In most of the cases, NPWT therapy was used as a "bridge to surgery therapy" in contaminated wounds. This term describes very well the approach which is used in our clinic. It was reported that NPWT facilitates a better mobilisation of patients compared to other strategies [13]. Depending on the extent of the wound, patients are able to sit, to perform pulmonary exercises and to walk. The change of the NPWT sponges in our hospital is always performed in OR, in most cases after two to three days, depending on the status of the wound and laboratory results. We have no standard scheme which is applied to every patient. The concept of NPWT has been applied and published in cases of post-sternotomy mediastinitis and showed promising results. Sjögren et al. [11] presented a 90-day mortality of 0% in a group of 53 pts. The group identified the time interval from cardiac surgery to diagnosis of deep sternal wound infection and the duration of wound therapy as independent predictors for late mortality. Prolonged use of NPWT appeared to be associated with recurrent infection and problems of the sternal wound in another publication with 21 patients with DSWI by Bapat et al. [14] In deep sternal infections after cardiac surgery they recommend NPWT therapy for a rather short period of time followed by early surgical closure if it seems sensible in the context [14].

In our hospital all patients suffering from DSI were treated with aggressive debridement of the wound and a subsequent NPWT therapy followed by pectoralis muscle plasty for secondary wound closure as previously described by our group [6].

In our experience wire removal and debridement should be undertaken as early as possible and should be performed in the operating theatre with a CBP capability and not as a bedside procedure. Sternal instability is the main cause for complications in postoperative therapies using negative pressure wound therapy. A restabilization of the sternum is absolutely necessary because of the possibility of mechanical alteration of the sternum that may lead to injuries of tissues underneath the sternum as observed in the described case of the patient who died.

For this complication of right ventricular rupture using NPWT only very few cases have been described until now [15]. In most of the cases the rupture occurred because of insufficient numbers of paraffin layers or insufficient placement of the layers. In other known cases where ventricular rupture occurred, it happened because of direct contact of the foam or the sternum with the underlying ventricle [16]. However, it might be speculated that some additional fatal events do not have been reported. These findings fit with our observation that the most common complication during the hospital stay of the analyzed patients is caused by the loss of sternal fixation or breaking of the cerclages. Other complications reported are few. There is one report about a retained piece of sponge [17], and reports about ventricle ruptures caused by direct contact of the sharp edges of the loosen sternum with tissues underneath as in our case [15].

In line with other publications [18-20] we recommend NPWT for infected wounds from small to large extent after cardiac surgery. To our knowledge there is no therapeutically concept which offers a similar "bridge to surgery" treatment with successful healing rates. In addition to this, patient mobilisation, pulmonary therapy and general nursing care improved in our hospital since the application of negative pressure wound therapy.

## Conclusion and future outlook

In line with the mentioned articles and reviews we come to the conclusion, that negative pressure wound treatment is a relatively new option for treatment of devastating wounds after sternotomy in cardiac surgery. As many institutions have presented their own approaches, it is important to find a strategy which may be used as a "standard NPWT approach" if identified in the future. It is therefore necessary to share our knowledge and experiences and to find an optimum treatment for these seriously ill patients in a consensus or algorithm e.g. proposed by the Lund University Hospital [21].

## Competing interests

The authors declare that they have no competing interests.

## Authors' contributions

IE drafted the manuscript, initiated the study and performed surgery, AM designed and drafted the manuscript and carried out the analysis of patients, DP participated in the development of the study, PV participated in the development of the surgical techniques and advised the content of the article, JE critically revised it for important intellectual content, AA analyzed and interpreted the data and performed the statistical analysis. All authors gave final approval of the version to be published.
